# Accelerated cisplatin and high-dose epirubicin with G-CSF support in patients with relapsed non-small-cell lung cancer: feasibility and efficacy

**DOI:** 10.1054/bjoc.2001.2013

**Published:** 2001-11

**Authors:** C Huisman, B Biesma, P E Postmus, G Giaccone, F M N H Schramel, E F Smit

**Affiliations:** Department of Pulmonary Diseases, University Hospital Vrije Universiteit, Amsterdam, The Netherlands; Department of Pulmonary Diseases, Bosch Medisch Centrum, Den Bosch, The Netherlands; Department of Medical Oncology, University Hospital Vrije Universiteit, Amsterdam, The Netherlands; Department of Pulmonary Diseases, St Antonius Hospital, Nieuwegein, The Netherlands

**Keywords:** non-small-cell lung cancer, chemotherapy, second-line, cisplatin, epirubicin

## Abstract

The purpose of this study is to determine whether it is feasible to administer high-dose epirubicin (135 mg m^−2^) combined with a fixed dose of cisplatin every 2 weeks with G-CSF support in patients with metastatic non-small-cell lung cancer (NSCLC). Subsequently, the efficacy of the recommended dose of this regimen was tested in a phase II study in patients with relapsed NSCLC. In the initial feasibility study at least 6 patients were entered at each of the 4 dose levels tested. A fixed dose of cisplatin 60 mg m^−2^was given. Epirubicin was administered at 120 mg m^−2^on dose level 1, 135 mg m^−2^on dose level 2 and 3 and 135 mg m^−2^on dose level 4. Patients treated at dose level 3 and 4 received G-CSF support on days 3–12. Cycles were repeated every 3 weeks on the first 3 dose levels and every 2 weeks on the fourth dose level. A total of 27 patients were then treated on dose level 4, which appeared to be feasible in the initial study. In the initial study, a total of 86 courses were administered. Haematological toxicity was the principal side effect. None of the patients encountered dose-limiting toxicity in the first course, which confirmed that epirubicin 135 mg m^−2^could be combined with cisplatin 60 mg m^−2^and accelerated by G-CSF support to a 14-day-schedule. In the subsequent phase II study with this schedule, 89 courses were administered. The relative dose intensity of cisplatin and epirubicin was 0.90 and 0.91, respectively. Myelosuppression was frequent with 70% and 63% of patients experiencing WHO grade III or IV leukocytopenia and thrombocytopenia, respectively. 6 cases of febrile neutropenia were observed, with 2 treatment-related deaths. Non-haematological toxicity consisted mainly of nausea and vomiting, which was grade III in 22% of patients. Renal toxicity grade I and II occurred in 37% and 4% of patients, respectively. 55% of these patients had received prior cisplatin-containing chemotherapy. On an intention-to-treat basis 9 partial responses were recorded in 27 patients (33%; 95% confidence interval, 15%–51%). Accelerated cisplatin and high-dose epirubicin with G-CSF support is a feasible and promising regimen in relapsed NSCLC. Myelosuppression limits the use of this regimen in the second-line setting to a selected group of patients with a good performance status. Since the activity of this regimen is encouraging, it is probably best studied in untreated patients.© 2001 Cancer Research Campaign  http://www.bjcancer.com


British Journal of Cancer (2001) 85(10), 1456–1461
© 2001 Cancer Research Campaign
doi: 10.1054/ bjoc.2001.2013, available online at http://www.idealibrary.com
http://www.bjcancer.com

### Accelerated cisplatin and high-dose epirubicin with G-CSF support in patients with relapsed non-small-cell lung cancer: feasibility and efficacy

**C Huisman[1,3], B Biesma[2], PE Postmus[1], G Giaccone[3], FMNH Schramel[4] and EF Smit[1]**

[1]Department of Pulmonary Diseases, University Hospital Vrije Universiteit, Amsterdam, The Netherlands; [2]Department of Pulmonary Diseases, Bosch Medisch Centrum, Den Bosch, The Netherlands; [3]Department of Medical Oncology, University Hospital Vrije Universiteit, Amsterdam, The Netherlands; [4]Department of Pulmonary Diseases, St Antonius Hospital, Nieuwegein, The Netherlands

**Summary** The purpose of this study is to determine whether it is feasible to administer high-dose epirubicin (135 mg $m^{-2}$) combined with a fixed dose of cisplatin every 2 weeks with G-CSF support in patients with metastatic non-small-cell lung cancer (NSCLC). Subsequently, the efficacy of the recommended dose of this regimen was tested in a phase II study in patients with relapsed NSCLC. In the initial feasibility study at least 6 patients were entered at each of the 4 dose levels tested. A fixed dose of cisplatin 60 mg $m^{-2}$ was given. Epirubicin was administered at 120 mg $m^{-2}$ on dose level 1, 135 mg $m^{-2}$ on dose level 2 and 3 and 135 mg $m^{-2}$ on dose level 4. Patients treated at dose level 3 and 4 received G-CSF support on days 3–12. Cycles were repeated every 3 weeks on the first 3 dose levels and every 2 weeks on the fourth dose level. A total of 27 patients were then treated on dose level 4, which appeared to be feasible in the initial study. In the initial study, a total of 86 courses were administered. Haematological toxicity was the principal side effect. None of the patients encountered dose-limiting toxicity in the first course, which confirmed that epirubicin 135 mg $m^{-2}$ could be combined with cisplatin 60 mg $m^{-2}$ and accelerated by G-CSF support to a 14-day-schedule. In the subsequent phase II study with this schedule, 89 courses were administered. The relative dose intensity of cisplatin and epirubicin was 0.90 and 0.91, respectively. Myelosuppression was frequent with 70% and 63% of patients experiencing WHO grade III or IV leukocytopenia and thrombocytopenia, respectively. 6 cases of febrile neutropenia were observed, with 2 treatment-related deaths. Non-haematological toxicity consisted mainly of nausea and vomiting, which was grade III in 22% of patients. Renal toxicity grade I and II occurred in 37% and 4% of patients, respectively. 55% of these patients had received prior cisplatin-containing chemotherapy. On an intention-to-treat basis 9 partial responses were recorded in 27 patients (33%; 95% confidence interval, 15%–51%). Accelerated cisplatin and high-dose epirubicin with G-CSF support is a feasible and promising regimen in relapsed NSCLC. Myelosuppression limits the use of this regimen in the second-line setting to a selected group of patients with a good performance status. Since the activity of this regimen is encouraging, it is probably best studied in untreated patients. © 2001 Cancer Research Campaign http://www.bjcancer.com

**Keywords**: non-small-cell lung cancer; chemotherapy; second-line; cisplatin; epirubicin

For patients with advanced non-small-cell lung cancer (NSCLC) treatment with chemotherapy results in symptom improvement and a small but significant survival benefit (Non-small cell lung cancer collaborative group, 1995). Cisplatin was an essential part of the combinations used and the other necessary drugs to achieve these effects were not identified. Therefore, cisplatin-containing combinations are currently considered the optimal treatment for advanced NSCLC patients with a good performance score (ASCO guidelines, 1997). Yet these regimens remain unsatisfactory because of treatment-related morbidity and limited effect on survival (Crinò et al, 1997). To improve upon these results, new drugs and new combinations are being extensively investigated in NSCLC. Given the short duration of response to first-line chemotherapeutic treatment, there is a need for non-cross-resistant chemotherapy in relapsing patients. Relapsing patients with a good performance score are candidates for clinical trials with novel agents or novel approaches, like dose-intensification (Huisman et al, 2000). The combination of cisplatin and epirubicin seems an attractive regimen to be explored for a dose–response relationship. For anthracyclines, a number of cancer models and phase I/II trials are indicative of such a relationship (Smit et al, 1992). 4″-epidoxorubicin (epirubicin) is a less cardiotoxic synthetic analogue of doxorubicin. When administered at doses up to 90 mg $m^{-2}$, single-agent epirubicin is not active in NSCLC (Kalman et al, 1983; Joss et al, 1984; Martoni et al, 1984; Meyers et al, 1986). In contrast, high-dose epirubicin (120–150 mg $m^{-2}$ every 4 weeks) resulted in response rates ranging from 19–36% in chemotherapy-naive patients with advanced NSCLC (Wils et al, 1990; Feld et al, 1992; Smit et al, 1992). Dose-limiting toxicity (DLT) is leukocytopenia of short duration, which may be overcome by the addition of haematopoietic growth factors.

The antitumour activity of single-agent high-dose epirubicin stimulated interest in further evaluation of this compound in combination with relatively non-myelosuppressive agents such as cisplatin. Dose intensification with the aid of haematopoietic growth factors was shown to be feasible for epirubicin single-agent therapy (Fountzilas et al, 1994) as well as in combination with paclitaxel (Lalisang et al, 2000) in breast cancer patients. In small-cell lung cancer patients the combination of cisplatin(100 mg $m^{-2}$) plus escalating doses of epirubicin (100–150 mg $m^{-2}$) was evaluated with the addition of granulocyte-macrophage colony-stimulating factor in case severe neutropenia was encountered (Rossel et al, 1994). The authors concluded that GM-CSF 250 μg $m^{-2}$ on

*Received 9 March 2001*
*Revised 3 June 2001*
*Accepted 5 July 2001*

*Correspondence to:* EF Smit

7–12 consecutive days did not have a myeloprotective effect when epirubicin was increased beyond 120 mg $m^{-2}$.

High-dose epirubicin in combination with cisplatin has been tested as first-line treatment in NSCLC patients. In a direct comparison high-dose epirubicin plus cisplatin was found to yield similar therapeutic results as cisplatin-vinorelbine (Martoni et al, 1998). In the second-line setting, standard dose epirubicin (60 mg $m^{-2}$) plus cisplatin (70 mg $m^{-2}$) was reported to obtain a 25% response rate in a group of 28 patients (Gridelli et al, 1992). All were pretreated with a non-platinum-based regimen and 64% had ECOG performance status 2.

Here we report on the results of dose-intensification of the cisplatin-epirubicin combination in patients with relapsed NSCLC. On the basis of a previous study (Smit et al, 1992) we considered the effective dose of epirubicin against NSCLC to be 135 mg $m^{-2}$. We tested whether epirubicin 135 mg $m^{-2}$ could be combined with cisplatin and accelerated by G-CSF support. Subsequently, the efficacy of this regimen was tested in patients with relapsed NSCLC.

### PATIENTS AND METHODS

### Patients

Patients with histologically or cytologically documented NSCLC were eligible for this study. Entry criteria included age range 18 to 76 years, WHO performance status 0–2 and a life expectancy of at least 2 months. Adequate haematological, renal and liver functions were required, defined as WBC ≥ 3.0 × $10^9$ $l^{-1}$, platelets ≥ 100 × $10^9$ $l^{-1}$, serum creatinine < 140 μmol $l^{-1}$ and/or creatinine clearance > 60 ml $min^{-1}$ (Cockroft–Gault formula) and bilirubin < 35 μmol $l^{-1}$. Prior cytotoxic treatment and radiotherapy were allowed provided that this had ended more than 4 weeks before entry into this study. Prior treatment with anthracyclines should not exceed a cumulative dose of 200 mg $m^{-2}$ in case of doxorubicin and 650 mg $m^{-2}$ in case of epirubicin. In the phase II study, patients with relapsed NSCLC after or progressive on previous chemotherapeutic treatment were included and patients were required to have bidimensionally measurable disease. Patients with symptomatic brain metastases, signs of cardiac failure or an active infection were excluded. Staging procedures consisted of chest X-ray and chest–abdomen CT scan. Brain CT, bone scan and abdominal ultrasound were performed when clinically indicated. The study was approved by the medical ethical committee at the Vrije Universiteit Amsterdam and all patients gave informed consent.

### Treatment

Cisplatin (Brystol–Myers Squibb, Woerden, the Netherlands) was diluted in 250 ml normal saline and administered intravenously over 60 minutes on day 1 of each course. Epirubicin (Pharmacia & Upjohn, Woerden, the Netherlands) dissolved in 250 ml normal saline was infused over 30 minutes on day 1 of each cycle prior to cisplatin. Forced intravenous hydration was administered with cisplatin. Prophylactic parenteral antiemetics consisted of dexamethasone plus a 5-HT3 receptor antagonist. A maximum of 6 cycles was planned. The dose of cisplatin was fixed throughout the study at 60 mg $m^{-2}$. The pre-defined dose levels of epirubicin in the phase I study are shown in Table 1. Dose-escalation of epirubicin was planned for, provided that none of the patients encountered DLT, defined as haematological toxicity WHO grade IV lasting for more than 5 days, neutropenic fever or any other toxicity exceeding WHO grade III except alopecia during the first course. G-CSF (Filgastrim, Amgen, Breda, the Netherlands) doses were 300 μg $day^{-1}$ for patients with a body weight < 60 kg and 480 μg $day^{-1}$ for patients with body weight > 60 kg administered on days 3–12 as a subcutaneous injection. At each dose level 6 patients were to be enrolled. We investigated whether epirubicin 135 mg $m^{-2}$ could be combined with cisplatin and accelerated by G-CSF support.

### Dose modification

The chemotherapy cycle was to be postponed for 1 week and a maximum of 2 weeks if WBC were < 3.0 × $10^9$ $l^{-1}$ and/or platelets < 100 × $10^9$ $l^{-1}$ at scheduled retreatment. Patients went off treatment if bone marrow recovery was still insufficient after 2 weeks of postponement.

Cisplatin was discontinued in case of creatinine clearance < 60 ml $min^{-1}$, oto-/neurotoxicity > WHO grade II. The dose of epirubicin was to be decreased from 120 mg $m^{-2}$ to 100 mg $m^{-2}$ and from 135 mg $m^{-2}$ to 120 mg $m^{-2}$ in case DLT was encountered. Epirubicin was discontinued if a second dose reduction of epirubicin was necessary.

### Treatment evaluation

Response to therapy was assessed every 2 courses according to WHO criteria. Responses were analysed on an intention-to-treat basis.

Toxicity was documented according to the WHO grading system. Patients were evaluable for toxicity if at least 1 cycle was completed. Investigations at entry consisted of medical history, physical examination including neurologic evaluation, WHO performance status, laboratory assessments (full blood count, electrolytes, liver enzymes, bilirubin, creatinine, creatinine clearance, total protein, albumin), electrocardiogram, tone and speech audiogram and tumour assessment (chest CT scan or other investigations that best defined disease extent and response evaluation). During treatment, a full blood count was measured at least weekly. Renal and liver function were determined before each cycle of chemotherapy. An audiogram was repeated every second cycle.

### RESULTS

### Feasibility study

From March 1997 to December 1997, 27 patients (22 men; median age 61 years; 85% ECOG 0 – 1) were enrolled at 4 dose levels in the initial feasibility study. 2 patients had stage IIIA disease, 11 stage IIIB and 14 stage IV. 14 patients had received previous therapy for NCSLC, consisting of chemotherapy in 12 patients (platinum-based in 2 patients), radiotherapy in 1 and combined radio-chemotherapy in 1 patient. None of the patients had received previous anthracyclines.

A total of 86 courses were administered. The relative dose intensity (RDI) of epirubicin was 0.98 (range 0.9–1) on dose level 1, 0.98 (range 0.91–1) on dose level 2, 0.98 (range 0.94–1) on dose level 3 and 0.94 (range 0.83–1) on dose level 4. For cisplatin these figures were 0.94 (range 0.6–1), 0.93 (range 0.6–1), 0.75 (range 0.2–1) and 0.94 (range 0.83–1), respectively.

All patients were evaluable for toxicity. Haematological toxicity was the principal side effect observed (Table 1). Anaemia led to red blood cell transfusions in 19 patients. The median number of

© 2001 Cancer Research Campaign

**Table 1** Dose levels and WHO haematologic toxicity (no. of patients) in the feasibility study

| | Cisplatin (mg m$^{-2}$) | Epirubicin (mg m$^{-2}$) | q (weeks) | G-CSF (5 µg kg$^{-1}$ day$^{-1}$) | Haemoglobin Parameter (WHO grade) | | | WBC Parameter (WHO grade) | | | Platelets Parameter (WHO grade) | | |
|---|---|---|---|---|---|---|---|---|---|---|---|---|---|
| | | | | | 0–2 | 3 | 4 | 0–2 | 3 | 4 | 0–2 | 3 | 4 |
| Dose level 1 (*n* = 8) | 60 | 120 | 3 | – | 6 | 2 | 0 | 1 | 3 | 4 | 5 | 3 | 0 |
| Dose level 2 (*n* = 6) | 60 | 135 | 3 | – | 4 | 2 | 0 | 1 | 3 | 2 | 3 | 2 | 1 |
| Dose level 3 (*n* = 6) | 60 | 135 | 3 | + | 6 | 0 | 0 | 2 | 3 | 1 | 5 | 0 | 1 |
| Dose level 4 (*n* = 7) | 60 | 135 | 2 | + | 5 | 2 | 0 | 3 | 2 | 2 | 4 | 1 | 2 |
| Total (*n* = 27) | | | | | 21 | 6 | 0 | 7 | 11 | 9 | 17 | 6 | 4 |

transfusion units was 2 per patient. The percentage of patients experiencing leukocytopenia WHO grade 3 or 4 at some time during treatment was 88% on dose level 1, 83% on dose level 2, 67% on dose level 3 and 57% on dose level, 4. Infectious complications were encountered in 9 patients, including 3 cases of febrile neutropenia (1 patient on dose level 1 after the second course, 2 patients on dose level 4 both after the third course). All patients recovered uneventfully from these infectious complications. Thrombocytopenia WHO grade 3 or 4 was noted in 38%, 50%, 17% and 43%, respectively. 1 patient on dose level 4 received a platelet transfusion. No haemorrhagic event due to thrombocytopenia occurred, however.

Non-haematological toxicity mainly consisted of nausea and vomiting, grade 1 or 2 in 24 out of 27 patients. All evaluable patients had alopecia ≥ WHO grade III. Grade I neurotoxicity occurred in 1 patient and in 2 patients audiometry showed asymptomatic hearing loss. In 5 patients creatinine clearance fell below 60 ml min$^{-1}$ resulting in cisplatin discontinuation. One of these patients was pretreated with cisplatin. Hypomagnesaemia ≥ WHO grade II was observed in 37% of patients.

Left ventricular ejection fraction was not assessed on a routine basis. In none of the patients were clinical manifestations of congestive heart failure (CHF) observed during treatment. However, 2 patients developed symptomatic cardiomyopathy at a later stage. 1 patient had received a cumulative epirubicin dose of 270 mg m$^{-2}$, followed by a total dose of 4000 cGy chest radiotherapy. 4 weeks thereafter the patient developed signs of CHF that favourably responded to cardiac medication. The other patient's medical history included general atherosclerosis and he had received a cumulative epirubicin dose of 675 mg m$^{-2}$. 6 weeks after the last epirubicin dose he was admitted because of dyspnoea due to severe left and right ventricular dysfunction. In spite of treatment the cardiac impairment proved irreversible and the patient died of CHF.

2 patients died during the study because of tumour progression. 1 patient treated on dose level one had disease progression after the first chemotherapy cycle and died because of acute renal failure. 7 patients did not have evaluable disease or treatment was stopped at their request after 1 course. 5 partial responses were achieved and 10 patients had disease stabilization. No complete responses were seen. Median survival time for all patients was 25 weeks (range 2–157 weeks).

None of the patients encountered DLT in the first course, which confirmed that epirubicin 135 mg m$^{-2}$ could be combined with cisplatin and accelerated by G-CSF support. Therefore, we proceeded with a phase II study to evaluate cisplatin 60 mg m$^{-2}$ and epirubicin 135 mg m$^{-2}$ plus G-CSF support in a 14-day schedule.

### Phase II study

### *Patient characteristics*

A total of 27 patients (18 male, 9 female) with a median age of 50 years (range: 36–75) were included between October 1997 and June 2000. 2 patients had stage IIIA disease, 8 stage IIIB and 17 stage IV. ECOG performance score was 0 in 11, 1 in 14 and 2 in 2 patients. 14 patients had adenocarcinoma. All patients had received prior chemotherapy for NSCLC, consisting of one cisplatin-based regimen in 59% of patients. 2 patients had received 2 prior chemotherapy regimens: pretreatment consisted of one platinum-based regimen in the 1 patient, whereas the other patient had not received any previous cisplatin at all. 14 out of 27 patients were considered as refractory, defined as relapsing within 3 months after or progressive on chemotherapy. The median time between last chemotherapy and entry into the study was 3 months (range 1–12 months). 6 patients had received radiotherapy on the primary tumour and 2 patients on supraclavicular lymphnodes. 2 patients had prior surgery.

### *Treatment administration*

A total of 89 courses were administered. The median number of courses administered to each patient was 3 (range 1 to 7). 1 patient received 6 courses of single-agent epirubicin because of impaired renal function after one course of cisplatin and epirubicin. 30% of patients received only one course. 1-week postponement of the next chemotherapy cycle due to thrombocytopenia occurred in 18% of courses. 2 patients went off study because of insufficient bone marrow recovery after 2 weeks treatment delay.

The relative dose intensity was 0.90 (range 0.1 to 1.00) for cisplatin and 0.91 for epirubicin (range 0.69 to 1.00). In 4 cases chemotherapy was stopped at the patient's request. 2 patients died during treatment due to progressive disease. One other early death occurred in a patient who was admitted because of pneumonia. The cause of death could not established since autopsy was not performed in this unexpected case. 2 treatment-related deaths occurred.

### *Response and survival*

In 6 patients treatment was discontinued before evaluation of response was performed: 3 patients died after the first cycle because of progressive disease and 3 patients received only one cycle at their own request. On an intention-to-treat basis 9 partial responses and no complete responses were recorded in 27 patients (33%; 95% confidence interval, 15–51%). 33% of patients had disease stabilization. Response was not evaluable in 6 patients. 5 out of 9 patients with partial responses had received prior platinum-containing therapy. 6 responders were considered

 © 2001 Cancer Research Campaign refractory to first-line treatment, defined as relapsing within 3 months after or progressive on chemotherapy.

Median follow-up was 22 weeks. One patient was lost to follow-up. The median duration of response was 28 weeks (range 6–55 weeks). At the time of analysis 6 patients were still alive. Median survival time for all patients was 27 weeks (range 1–65 weeks).

### *Toxicity*

All patients were evaluable for toxicity. Haematological toxicity was the principal side effect (Table 2). 2 patients were not evaluable for anaemia because of concomitant use of erythropoietin. 20% of patients experienced grade III anaemia and 20 patients received red blood cell transfusions. The median number of transfusion units was 3 per patient. 70% and 63% of patients experienced grade III or IV leukocytopenia and thrombocytopenia, respectively. Platelet transfusions were necessary in 6 patients, 1 of whom experienced epistaxis and 1 had haemorrhagic discharge from his tracheostoma. Infectious episodes occurred in 3 patients. Moreover, 6 cases of febrile neutropenia were observed in 6 patients. Whereas 4 patients with febrile neutropenia recovered uneventfully with intravenous antibiotics, 2 patients with febrile neutropenia in their first course developed sepsis and died in spite of antibiotic treatment.

Non-haematological toxicity (Table 3) consisted mainly of nausea and vomiting, which was severe (grade III) in 22% of patients. Alopecia was universal. Renal toxicity grade I and II occurred in 37% and 4% of patients, respectively. 55% of these patients had received prior cisplatin-containing chemotherapy. Hyponatriemia occurred in 2 patients and was symptomatic in 1 of them. This patient had previously been treated with platinum. Asymptomatic hypomagnesaemia ≥ WHO grade II was observed in 50% of patients. No cardiac complications were observed during the study.

### DISCUSSION

This study was aimed to assess the feasibility and efficacy of high-dose epirubicin in combination with cisplatin in patients with advanced NSCLC who relapsed after or progressed on first-line chemotherapy. The results of our initial feasibility study demonstrated that epirubicin doses could be escalated to 135 mg $m^{-2}$ every 2 weeks provided that G-CSF support was added. Further dose escalation was not performed, since it has been reported that treatment becomes more toxic and impracticable at doses of 150 mg $m^{-2}$ or above (Martoni et al, 1991).

Although chemotherapy is an established treatment for advanced NSCLC, data on its therapeutic activity in relapsed NSCLC are still very limited. Response rates in relapsed NSCLC are poor and the first agent that has been approved for this indication in several countries, docetaxel, obtained a response rate of 7–11% in randomized trials (Fosella et al, 2000; Shepherd et al, 2000). Against this background, our study of accelerated cisplatin and high-dose epirubicin with G-CSF support suggests relevant activity of this regimen in relapsed NSCLC.

Criteria for the selection of patients for second-line chemotherapy in NSCLC have not been clearly defined yet. The need for definitions of patient groups is underlined by the upcoming difficulty to test new drugs in previously untreated patients. The second-line setting may be most suitable for testing new drugs or drug combinations (Huisman et al, 2000). However, haematological toxicities may be more pronounced in this patient population.

**Table 2** Phase II: WHO haematologic toxicity (no. of patients)

| | Worst WHO grade | | | | | | | | | |
|---|---|---|---|---|---|---|---|---|---|---|
| | 0 | | 1 | | 2 | | 3 | | 4 | |
| Parameter | No. | % | No. | % | No. | % | No. | % | No. | % |
| Haemoglobin | 0 | 0 | 5 | 20 | 15 | 60 | 5 | 20 | 0 | 0 |
| WBC | 1 | 4 | 5 | 19 | 2 | 7 | 6 | 22 | 13 | 48 |
| Platelets | 0 | 0 | 7 | 26 | 3 | 11 | 7 | 26 | 10 | 37 |

**Table 3** Phase II: WHO nonhaematologic toxicity (no. of patients)

| | Worst WHO grade | | | | | | | | | |
|---|---|---|---|---|---|---|---|---|---|---|
| | 0 | | 1 | | 2 | | 3 | | 4 | |
| Parameter | No. | % | No. | % | No. | % | No. | % | No. | % |
| Nausea/vomiting | 3 | 11 | 12 | 44 | 6 | 22 | 6 | 22 | 0 | 0 |
| Hepatic | | | | | | | | | | |
| Alkaline phosphatase | 22 | 82 | 5 | 18 | 0 | 0 | 0 | 0 | 0 | 0 |
| Transaminases | 26 | 96 | 1 | 4 | 0 | 0 | 0 | 0 | 0 | 0 |
| Bilirubin | 27 | 100 | 0 | 0 | 0 | 0 | 0 | 0 | 0 | 0 |
| Renal | 16 | 59 | 10 | 37 | 1 | 4 | 0 | 0 | 0 | 0 |
| Hypomagnesaemia | 5 | 19 | 8 | 31 | 8 | 31 | 2 | 8 | 3 | 11 |
| Neurotoxicity | 26 | 96 | 1 | 4 | 0 | 0 | 0 | 0 | 0 | 0 |
| Ototoxicity | 26 | 96 | 1 | 4 | 0 | 0 | 0 | 0 | 0 | 0 |

*© 2001 Cancer Research Campaign*

In the present study, responses were obtained both in sensitive (defined as relapsing more than 3 months after prior chemotherapy) and refractory patients. There was no clear correlation between a response to first-line and to second-line therapy. As reviewed elsewhere (Huisman et al, 2000), conflicting results have been reported about the influence of response to prior platinum-containing chemotherapy. In the current study, 5 out of 9 responders had received previous platinum-containing chemotherapy. One of them was progressive on a first-line platinum-containing regimen, whereas the duration of response to first-line therapy of the 4 other patients ranged from 3 to 10 months. Moreover, neither neurotoxicity nor nephrotoxicity were severe in the subgroup of patients, which emphasizes the feasibility to administer platinum-containing chemotherapy to patients who were pretreated with platinum. In our phase II study severe myelosuppression was frequent, with 6 cases of febrile neutropenia and 2 treatment-related deaths, limiting its use in the second-line setting to a selected group of patients with a good performance status. 15% of courses were postponed due to thrombocytopenia. It is important to consider the effect of frequent dose delay due to insufficient bone marrow recovery on the dose-intensity intended. In the phase II part of our study, the relative dose intensities of cisplatin and epirubicin were 0.93 and 0.92, respectively. In general, the thrombocytopenia was readily reversible and no serious haemorrhagic events occurred.

Non-haematological toxicity was generally mild. Renal toxicity was not more evident in patients who had received prior cisplatin-containing chemotherapy, probably due to the moderate dose of platinum used in this schedule. No clinical manifestations of congestive heart failure were observed during treatment or follow-up phase of the phase II study. Since it has been demonstrated that a cumulative dose of 1000 mg $m^{-2}$ epirubicin is infrequently associated with cardiac toxicity (Smit et al, 1992), we do not recommend routine evaluation of left ventricular function for this schedule.

Whereas G-CSF support (300 – 480 μg $day^{-1}$) was valuable at an epirubicin dose of 135 μg $m^{-2}$ in the current study, others have concluded that GM-CSF 250 μg $m^{-1}$ did not have a myeloprotective effect when epirubicin was increased beyond 120 mg $m^{-2}$ (Rossel et al, 1994). However, a different protocol of GM-CSF administration was applied in this study of small-cell lung cancer patients, since GM-CSF was administered only to those patients who developed febrile neutropenia in a previous cycle. Moreover, absence of myeloprotection provided by GM-CSF was most pronounced at epirubicin doses of 150 mg $m^{-2}$. At the 130 mg $m^{-2}$ epirubicin dose effective myeloprotection was achieved in 2 out of 3 patients, whereas the patient in whom GM-CSF support was not effective had already received 5 previous cycles of chemotherapy. The authors do not describe the effect of GM-CSF in 3 patients who encountered febrile neutropenia in their second, fifth and eighth cycle at an epirubicin dose of 140 mg $m^{-2}$. Finally, at an epirubicin dose of 150 mg $m^{-2}$ myelosuppression was clearly severe and could not be prevented by GM-CSF administration.

The second-line activity of accelerated cisplatin and high-dose epirubicin with G-CSF support generates interest in evaluating this schedule as a first-line regimen for advanced NSCLC. Haematological toxicity was severe but manageable and may well be better tolerated in untreated patients. Moreover, especially for patients receiving combined modality treatment, a chemotherapy schedule that can be completed within a short time period is an attractive treatment option. Martoni et al reported interesting results from a trial with a comparable but not accelerated regimen in the first-line setting (Martoni et al, 1992). They treated 37 patients with advanced NSCLC with cisplatin (60 mg $m^{-2}$) and epirubicin (120 mg $m^{-2}$) on day 1 every 28 days and observed a 54% response rate with median response duration of 10 months and median survival of 9 months. They attributed their high response rate to doubling of the epirubicin dose as compared to an earlier study that achieved an 18% response rate with epirubicin 60 mg $m^{-2}$ and cisplatin 50 mg $m^{-2}$ (Lelli et al, 1986). Results from a consecutive multicentre randomized clinical trial confirmed that cisplatin plus high-dose epirubicin is an active first-line regimen in stage III A–IV NSCLC, with a 32% response rate and a 42% 1-year survival (Martoni et al, 1998). We feel the combination of these drugs accelerated by G-CSF should be investigated further for its role as neoadjuvant chemotherapy. Although epirubicin is less cardiotoxic than doxorubicin, patients receiving subsequent radiotherapy should be monitored carefully.

In conclusion, accelerated cisplatin and high-dose epirubicin with G-CSF support is feasible and promising in relapsed NSCLC. Myelosuppression limits its use in the second-line setting to a selected group of patients with a good performance status. Since the activity of this regimen is encouraging, it is probably best studied in untreated patients.

### REFERENCES

ASCO (1997) Clinical practice guidelines for the treatment of unresectable non-small cell lung cancer. *J Clin Oncol* **15**: 2996–3018

Crinò L, Scagliotti G, Marangolo M et al (1997) Cisplatin-gemcitabine combination in advanced non-small cell lung cancer: a phase II study. *J Clin Oncol* **15**: 297–303

Feld R, Wierzbicki R, Walde PLD et al (1992) Phase I-II study of high-dose epirubicin in advanced non-small cell lung cancer. *J Clin Oncol* **10**: 297–303

Fossella FV, DeVore R, Kerr R et al (2000) Randomized phase III trial of docetaxel versus vinorelbine or ifosfamide in patients with non-small cell lung cancer previously treated with platinum-containing chemotherapy. *J Clin Oncol* **18**: 2354–2362

Fountzilas G, Skarlos D, Giannakakis T et al (1994) Intensive chemotherapy with high-dose epirubicin every 2 weeks and prophylactic administration of filgastrim in advanced breast cancer. *Eur J Cancer* **30**: 965–969

Gridelli C, Airoma G, Incoronato P et al (1992) Mitomycin C plus vindesine or cisplatin plus epirubicin in previously treated patients with symptomatic advanced non-small cell lung cancer. *Cancer Chemother Pharmacol* **30**: 212–214

Huisman C, Smit EF, Giaccone G and Postmus PE (2000) Second-line chemotherapy in relapsing or refractory non-small cell lung cancer patients – a review. *J Clin Oncol* **18**: 3722–3730

Joss RA, Hansen HH, Hansen M et al (1984) Phase II clinical trial of epirubicin in advanced squamous, adeno-and large cell carcinoma of the lung. *Eur J Cancer Clin Oncol* **20**: 495–499

Kalman LA, Kris MG, Gralla RJ et al (1983) Phase II trial of 4'-epidoxorubicin in patients with non-small cell lung cancer. *Cancer Treat Rep* **67**: 591–592

Lalisang RI, Voest EE, Wils JA et al (2000) Dose-dense epirubicin and paclitaxel with G-CSF: a study of decreasing intervals in metastatic breast cancer. *Br J Cancer* **82**: 1914–1919

Lelli G, Farris A, Casadio M et al (1986) 4'-epidoxorubicin and cisdiamminedichloroplatinum in advanced non-small cell bronchogenic carcinoma: a phase II study. *J Exp Clin Cancer Res* **5**: 185–190

Martoni A, Giovanni M and Tomasi L (1984) A phase II clinical trial of 4'-epidoxorubicin in advanced solid tumours. *Cancer Chemother Pharmacol* **12**: 179–182

Martoni A, Melotti B, Guaraldi M et al (1991) Activity of high-dose epirubicin in advanced non-small cell lung cancer. *Eur J Cancer* **27**: 1231–1234

Martoni A, Guaraldi M, Casadio L et al (1992) A phase II study of high-dose epirubicin plus cisplatinum in advanced non-small cell lung cancer. *Ann Oncol* **3**: 864–866

Martoni A, Guaraldi M, Piana E et al (1998) Multicenter randomized clinical trial on high-dose epirubicin plus cisplatinum versus vinorelbine plus cisplatinum in advanced non-small cell lung cancer. *Lung Cancer* **22**: 31–38

*© 2001 Cancer Research Campaign*

Meyers FJ, Cardiff RD, Quadro R et al (1986) Epirubicin in non-oat cell lung cancer-response rates and the importance of immunopathology: a northern California oncology group study. *Cancer Treat Rep* 1986; **70**: 805–806

Non-small cell lung cancer collaborative group (1995) Chemotherapy in non-small cell lung cancer: a meta-analysis using updated data on individual patients from 52 randomized trials. *BMJ* **311**: 899–909

Rossel R, Gómez-Codina J, Anton A et al (1994) Escalating high-dose epirubicin plus cisplatin in small cell lung cancer with granulocyte-macrophage colony-stimulating factor use when appropriate. *Semin Onc* **21** (**Suppl 1**): 48–53

Shepherd FA, Dancey J, Ramlau R et al (2000) Prospective randomized trial of docetaxel versus best supportive care in patients with non-small cell lung cancer previously treated with platinum-based chemotherapy. *J Clin Oncol* **18**: 2095–2103

Smit EF, Berendsen HH, Piers DA et al (1992) A phase II study of high dose epirubicin in unresectable non small cell lung cancer. *Br J Cancer* **65**: 405–408

Wils J, Utama I, Sala L et al (1990) Phase II study of high dose epirubicin in non-small cell lung cancer. *Eur J Cancer* **26**: 1140–1141

*© 2001 Cancer Research Campaign*
